# Magnesium Sulfate Prevents Placental Ischemia-Induced Increases in Brain Water Content and Cerebrospinal Fluid Cytokines in Pregnant Rats

**DOI:** 10.3389/fnins.2016.00561

**Published:** 2016-12-08

**Authors:** Linda W. Zhang, Junie P. Warrington

**Affiliations:** Department of Physiology and Biophysics, University of Mississippi Medical CenterJackson, MS, USA

**Keywords:** cerebrospinal fluid, eclampsia, placental ischemia, cytokines, chemokines

## Abstract

Magnesium sulfate (MgSO_4_) is the most widely used therapy in the clinic to prevent the progression of preeclampsia, a hypertensive disorder of pregnancy, to eclampsia. Eclampsia, manifested as unexplained seizures and/or coma during pregnancy or postpartum, accounts for ~13% of maternal deaths worldwide. While MgSO_4_ continues to be used in the clinic, the mechanisms by which it exerts its protective actions are not well understood. In this study, we tested the hypothesis that MgSO_4_ protects against placental ischemia-induced increases in brain water content and cerebrospinal fluid cytokines. To test this hypothesis, MgSO_4_ was administered via mini-osmotic pump (60 mg/day, i.p.) to pregnant and placental ischemic rats, induced by mechanical reduction of uterine perfusion pressure, from gestational day 14–19. This treatment regimen of MgSO_4_ led to therapeutic level of 2.8 ± 0.6 mmol/L Mg in plasma. MgSO_4_ had no effect on improving placental ischemia-induced changes in mean arterial pressure, number of live fetuses, or fetal and placental weight. Placental ischemia increased, while MgSO_4_ prevented the increase in water content in the anterior cerebrum. Cytokine and chemokine levels were measured in the cerebrospinal fluid using a multi-plex assay. Results demonstrate that cerebrospinal fluid, obtained via the cisterna magna, had reduced protein, albumin, interleukin (IL)-17A, IL-18, IL-2, eotaxin, fractalkine, interferon gamma, vascular endothelial growth factor (VEGF), and macrophage inflammatory protein (MIP)-2 following MgSO_4_ treatment. These data support the hypothesis that MgSO_4_ offers neuroprotection by preventing placental ischemia-induced cerebral edema and reducing levels of cytokines/chemokines in the cerebrospinal fluid.

## Introduction

Magnesium sulfate (MgSO_4_) is the main course of treatment for preeclampsia patients with severe symptoms and is often used to prevent the progression of the disorder from preeclampsia to eclampsia. Preeclampsia complicates about 5–8% of pregnancies in the United States (Saftlas et al., 1990) and close to 20% of African American pregnancies (Mostello et al., 2002). Preeclampsia is characterized by new-onset hypertension with proteinuria or in the absence of proteinuria, low platelet count, renal insufficiency, impaired liver function, pulmonary edema, or cerebral or visual symptoms manifesting after the 20th week of gestation (American College of Obstetricians & Gynecologists and Task Force on Hypertension in Pregnancy, 2013). Preeclampsia affects multiple organs including the kidney, liver, and brain. It should be noted that of all preeclampsia/eclampsia-related deaths, cerebrovascular events are the cause in ~40% of cases (MacKay et al., 2001). Additional evidence that the cerebral vasculature is affected in preeclampsia is provided by the common manifestations of neurological symptoms ranging from headaches to seizures (in the case of eclampsia) (Chakravarty and Chakrabarti, 2002) in patients. While MgSO_4_ is a common treatment, early delivery of the fetus and removal of the placenta is the only method to reverse the disorder (Sibai et al., 2005; Sibai, 2006). Thus, novel therapies for the treatment of preeclampsia are needed, and the mechanisms by which MgSO_4_ exerts its beneficial effects need to be elucidated.

The findings of blood-brain barrier (BBB) disruption and cerebral edema formation using various imaging modalities, such as MRI and CT scans (Apollon et al., 2000; Demirtaş et al., 2005; Aygün et al., 2010) demonstrate that the cerebral vasculature is affected in preeclampsia patients. Further evidence of BBB disruption has been provided using an isolated vessel preparation that showed that plasma from preeclampsia patients increases cerebral venous permeability (Amburgey et al., 2010; Schreurs and Cipolla, 2013; Schreurs et al., 2013). While the BBB regulates the exchange of substances between the blood and the brain, the blood-cerebrospinal fluid (CSF)-barrier, formed primarily by choroid plexus epithelial cells (ependymal cells) or meningothelial cells, regulates the substances exchanged between the blood and CSF. A recent study from our lab demonstrated that placental ischemic rats have increased inflammatory cytokines in the CSF (Warrington, 2015), suggesting that there is increased blood-CSF-barrier permeability or increased production and clearance of cytokines from the extracellular space to the CSF in response to placental ischemia. Whether MgSO_4_ has effects on placental ischemia-induced increases in CSF cytokines or the blood-CSF-barrier permeability is not known.

We utilized the rat model of placental ischemia, induced by reducing uterine perfusion pressure (RUPP) to mimic the clinical condition of preeclampsia. The placental ischemic model has numerous similar characteristics to preeclampsia patients. For example, like preeclampsia patients, placental ischemic rats have increased arterial blood pressure with or without proteinuria (Alexander et al., 2001). Additionally, placental ischemia leads to increased circulating and placental levels of inflammatory cytokines (LaMarca et al., 2005; Gadonski et al., 2006), and increased anti-angiogenic factors (Gilbert et al., 2007, 2009), along with similar cerebrovascular changes as preeclampsia patients. Recent studies have shown marked impairment in cerebral blood flow autoregulation, increased BBB permeability, (Warrington et al., 2014) impaired cerebrovascular myogenic tone, and cerebral edema (Ryan et al., 2011) in response to placental ischemia. Importantly, a recent study by Johnson et al. demonstrated increases in BBB permeability and microglial activation following placental ischemia and high cholesterol to mimic severe preeclampsia (Johnson et al., 2014). While the study assessed changes in brain water content in the posterior cerebrum, there were no reports of the effects of MgSO_4_ treatment on edema formation in the anterior cerebrum. Thus, since the placental ischemia model is characterized by edema in the anterior cerebrum (Warrington et al., 2014, 2015), we determined whether MgSO_4_ would improve placental ischemia-induced cerebral edema in the anterior and posterior cerebrum.

The purpose of this study was to assess whether MgSO_4_, administered during the third trimester (from gestational day 14–19), leads to improvements in blood pressure, fetal outcome, brain water content, blood-CSF-barrier permeability, and CSF cytokine/chemokine levels following placental ischemia. While a recent study showed beneficial effects of acute MgSO_4_ treatment on seizure activity and microglial activation in a rat model of severe preeclampsia (placental ischemia plus high cholesterol diet) (Johnson et al., 2014), this is the first study to explore whether chronic administration of MgSO_4_ can improve the general characteristics of blood pressure, fetal demise, and CSF cytokines in rats exposed to placental ischemia alone.

## Materials and methods

### Animals

Timed pregnant Sprague-Dawley (CD) rats were obtained from Charles Rivers Laboratories and arrived at the Lab Animal Facilities at the University of Mississippi Medical Center on gestational day 11. All animal procedures were approved by the Institutional Animal Care and Use Committee at the University of Mississippi Medical Center. The rats were maintained on a 12 h light/ 12 h dark cycle and fed standard rodent chow and water *ad libitum*.

### Reduced uterine perfusion pressure (RUPP) model of placental ischemia

To induce placental ischemia, silver clips were surgically inserted around the abdominal aorta, below the kidneys, and each of the uterine artery branches from the ovary on day 14 of gestation as described previously. This procedure reduces blood flow to the utero-placental unit by ~40% in pregnant rats (Granger et al., 2006). Animals were randomly assigned to normal pregnant (NP) or placental ischemia (RUPP) groups.

### Magnesium sulfate infusion

On gestational day 14, normal pregnant and placental ischemic rats were implanted with mini-osmotic pumps to deliver 60 mg/day MgSO_4_ (diluted in 0.9% saline solution) via the intraperitoneal cavity. The resulting groups were NP (no MgSO_4_), RUPP (no MgSO_4_), NP + MgSO_4_, and RUPP + MgSO_4_. Using a commercially available kit (abcam, ab102506), Mg levels in the plasma were 2.8 ± 0.6 mmol/L at the end of the study (gestational day 19). Thus, therapeutic doses of Mg were obtained.

### Measurement of mean arterial pressure (MAP) and fetal outcomes

On gestational day 18, rats were surgically instrumented with carotid catheters for the measurement of mean arterial pressure. On GD19, following at least 30 min acclimation to the restrainer cages, blood pressure was recorded for 30 min. The mean arterial pressure was then obtained using LabChart software. The uterine horn was examined for determination of the number of live and resorbed fetuses and the placentas and pups were removed and weighed.

### Collection of cerebrospinal fluid

On gestational day 19, rats were anesthetized using isoflurane and secured on a stereotaxic frame and cerebrospinal fluid was obtained via the cisterna magna using a butterfly needle and syringe. CSF samples were flash frozen in liquid nitrogen and stored at −80°C until processing.

### Determination of brain water content

Following CSF collection, brains were harvested, cerebellum dissected off, and cerebrum divided along the midline into two hemispheres. Half of the brain was used for determination of brain water content and was further separated into anterior and posterior cerebrum by cutting along the middle cerebral artery. Brain tissue anterior to the middle cerebral artery was designated as anterior cerebrum while tissue posterior to the middle cerebral artery (without the cerebellum) was designated as posterior cerebrum. The brain regions were weighed and then dried in an oven at 60°C for 72 h, after which dry weight was obtained. Brain water content was then calculated as a percentage: [(wet weight-dry weight)/wet weight] × 100.

### Measurement of CSF protein and albumin/blood-CSF-barrier permeability

Cerebrospinal fluid (CSF) samples were thawed and total protein was measured using the Bicinchoninic acid (BCA) kit (ThermoFisher, 23225). Albumin content in the CSF was measured using a Rat Albumin ELISA kit (GenWay BioTech, GWB-1B2B4B) using a dilution factor of 1:1000. Kits were run according to the manufacturer's directions.

### Measurement of CSF cytokine/chemokine concentration

Cerebrospinal fluid (CSF) samples were thawed and assayed using the rat cytokine/chemokine magnetic bead panel (Milliplex MAP kit, EMD Millipore, RECYMAG65K27PMX) which included 27 cytokines/chemokines. CSF samples were assayed undiluted following manufacturer's directions. Cytokine/chemokine concentration was determined from a standard curve generated for each analyte.

### Statistical analysis

Two-Way Analysis of Variance (group–NP and RUPP vs. treatment–no MgSO_4_ or MgSO_4_ treated) was used to determine differences amongst the groups followed by Fisher's LSD *post-hoc* test. Comparisons were considered statistically significant if *p* < 0.05.

## Results

### MgSO_4_ has no effect on placental ischemia-induced increases in blood pressure or fetal demise

The effects of placental ischemia and MgSO_4_ treatment on the general characteristics of the rats are summarized in Table 1. Placental ischemia resulted in a significant increase in MAP and number of resorbed pups. MgSO_4_ treatment had no effect on placental ischemia-induced increases in MAP or number of resorbed pups. Placental ischemia led to a significant decrease in the number of live fetuses and pup weight. MgSO_4_ had no effect on placental ischemia-induced decreases in number of live fetuses or pup weight. MgSO_4_ treatment reduced placenta weight in the placental ischemic group. In normal pregnant rats, MgSO_4_ treatment had no effect on any of the parameters measured.

**Table 1:** **Summary of the general characteristics of pregnant rats**.

**Characteristics**	**NP (*N* = 6)**	**RUPP (*N* = 6)**	**NP + MgSO_4_ (*N* = 9)**	**RUPP + MgSO_4_ (*N* = 11)**
MAP (mmHg)	102±2	115±3^*^^†^	100±4	114±2^*^^†^
No. of Live Pups	14±0	7±2^*^^†^	13±1	6±1^*^^†^
No. of Resorbed Pups	0±0	6±2^*^^†^	1±0	7±1^*^^†^
Pup Weight (g)	2.47±0.06	2.24±0.09^*^	2.27±0.06	2.16±0.05^*^
Placenta Weight (g)	0.47±0.02	0.46±0.01	0.46±0.01	0.38±0.02^*^^†^

* p < 0.05 vs. NP;

† *p < 0.05 vs. NP + MgSO_4_ group*.

### MgSO_4_ prevented placental ischemia-induced increases in brain water content

**Figure 1:**
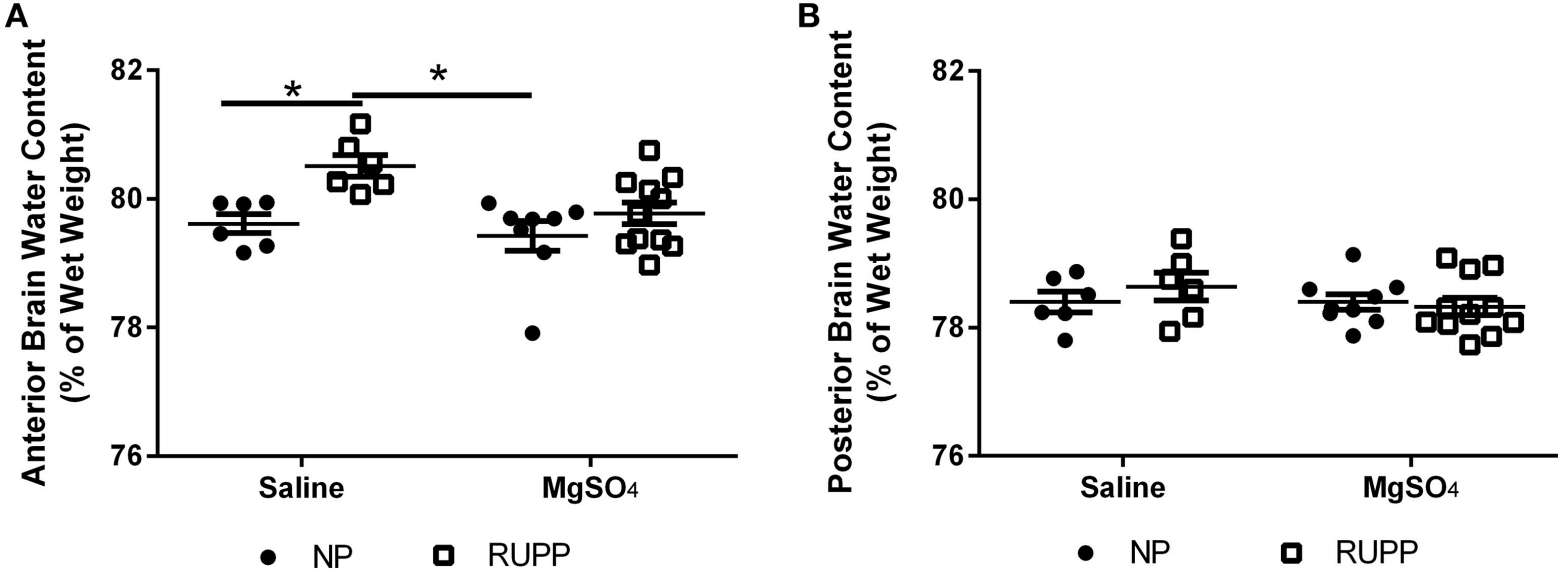
**Changes in brain water content in response to placental ischemia and MgSO_**4**_ treatment. (A)** Placental ischemia (RUPP) increases anterior cerebrum water content in pregnant rats while MgSO_4_ prevents the increase in brain water content in response to placental ischemia. No change in water content in: **(B)** posterior cerebrum. Bars represent Mean ± SEM. ^*^p < 0.05. *N* = 6–11 per group/treatment. NP, normal pregnant; RUPP, reduced uterine perfusion pressure.

To determine whether MgSO_4_ prevents placental ischemia-induced cerebral edema, brain water content was measured. Anterior brain water content increased in response to placental ischemia (Figure 1A) from 79.6 ± 0.1% in the pregnant group to 80.5 ± 0.3% (*p* < 0.01). MgSO_4_ prevented the increase in brain water content in response to placental ischemia (79.8 ± 0.2%, *p* = 0.01 compared to RUPP). In normal pregnant rats, MgSO_4_ treatment had no effect on brain water content (79.4 ± 0.2%). There were no changes in brain water content in the posterior cerebrum in response to placental ischemia or MgSO_4_ treatment (Figure 1B).

### MgSO_4_ reduces protein and albumin concentrations in the cerebrospinal fluid

**Figure 2:**
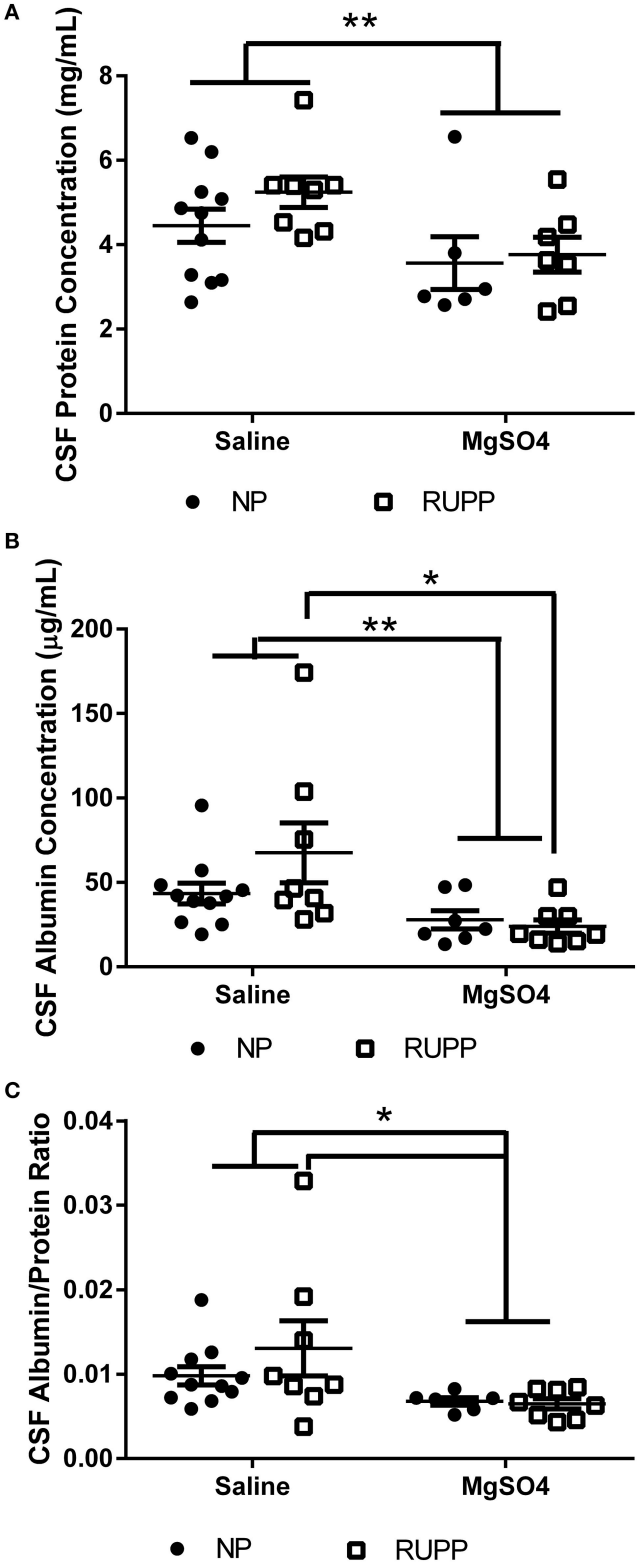
**Changes in cerebrospinal fluid protein and albumin content**. MgSO_4_ treatment decreases **(A)** CSF protein concentration, **(B)** CSF albumin content, and **(C)** The ratio of albumin/protein. Bars represent Mean ± SEM. ^*^p < 0.05, ^**^p < 0.01. *N* = 6–11 per group/treatment. NP, normal pregnant; RUPP, reduced uterine perfusion pressure.

Placental ischemic rats had 5.2 ± 0.4 mg/mL protein compared to 4.5 ± 0.4 mg/mL in the normal pregnant group (Figure 2A). MgSO_4_ reduced protein content in the CSF in the placental ischemic group to 3.6 ± 0.4 mg/mL; *p* = 0.03. MgSO4 treatment had no effect on CSF protein concentration in the normal pregnant group (3.6 ± 0.6 mg/mL). CSF albumin concentration was 43.5 ± 6.2 μg/mL in the normal pregnant saline treated group and 67.5 ± 17.7 μg/mL in the placental ischemic group (*p* = 0.076; Figure 2B). MgSO_4_ significantly reduced albumin concentration in the placental ischemic group (23.8 ± 4.0 μg/mL; *p* < 0.01) but had no effect on CSF albumin concentration in normal pregnant rats (27.9 ± 5.4 μg/mL). Cerebrospinal fluid albumin/protein ratio was calculated for each of the animals (Figure 2C). The albumin/protein ratio in the normal pregnant rats was 0.010 ± 0.001 compared to 0.013 ± 0.003 in the placental ischemia (RUPP) non-treated group. MgSO_4_ treatment significantly reduced the albumin/protein ratio in the placental ischemic group to 0.006 ± 0.0001; *p* = 0.02 but had no effect in the normal pregnant group (0.007 ± 0.0000).

### MgSO_4_ reduces cerebrospinal fluid cytokine levels

To determine whether MgSO_4_ leads to a reduction in CSF cytokines, a multi-plex array was used to measure 27 different cytokine/chemokines in the CSF. The concentrations are summarized in Table 2. Two-way analysis of variance revealed significant increases in CSF eotaxin, IL-2, and IL-18 in response to placental ischemia. MgSO_4_ treatment resulted in significant decreases in CSF eotaxin, fractalkine, GRO/KC/CINC, interferon gamma, IL-2, IL-17, IL-18, MIP-2, and VEGF. MgSO_4_ treatment of normal pregnant rats had no effect on CSF cytokines/chemokines except for MIP-2 where it was decreased.

**Table 2:** **Summary of cytokines/chemokines in the CSF of rats subjected to placental ischemia and/or MgSO_**4**_**.

**Cytokine/Chemokine**	**NP (*N* = 7)**	**RUPP (*N* = 6)**	**NP + MgSO_4_ (*N* = 6)**	**RUPP + MgSO_4_ (*N* = 7)**
EGF (pg/mL)	ND	ND	ND	ND
Eotaxin	20.7 ± 2.9	31.3 ± 1.4^*^	17.0 ± 3.8^‡^	19.7 ± 4.5^‡^
Fractalkine	36.2 ± 4.2	45.0 ± 3.4	31.3 ± 4.3^‡^	29.8 ± 3.9^‡^
G-CSF	16.6 ± 1.7	15.6 ± 2.4	11.8 ± 2.3	17.5 ± 1.8
GM-CSF	184.5 ± 25.7	212.1 ± 37.0	159.9 ± 39.2	190.9 ± 34.1
GRO/KC/CINC	239.7 ± 30.6	243.9 ± 14.2	186.6 ± 32.0	163.05 ± 15.8^*^^‡^
Interferon Gamma	42.4 ± 6.5	61.7 ± 9.6	31.5 ± 7.9^‡^	30.3 ± 8.6^‡^
Interleukin-10	12.4 ± 3.2	20.9 ± 4.5	10.9 ± 2.6 (5)	17.3 ± 5.9
Interleukin-12p70	ND	23.47 (2)	ND	ND
Interleukin-13	4.3 ± 2.0 (5)	7.8 ± 3.7	4.7 ± 2.6 (3)	6.1 ± 1.9 (5)
Interleukin-1 alpha	11.2 ± 1.0 (2)	7.3 ± 2.3 (5)	6.1 ± 2.1 (3)	6.4 ± 3.3 (3)
Interleukin-1 beta	25.1 ± 8.4	28.7 ± 5.2	14.8 ± 4.3	18.4 ± 5.1
Interleukin-2	40.7 ± 4.8	59.5 ± 9.5^*^	35.8 ± 5.2^‡^	40.3 ± 5.7^‡^
Interleukin-4	12.7 ± 2.3	16.0 ± 3.7	13.3 ± 2.1	13.4 ± 2.1
Interleukin-5	25.0 ± 2.9	29.8 ± 5.2	19.8 ± 4.5	25.3 ± 3.4
Interleukin-6	343.2 ± 58.4	367.5 ± 83.3	301.8 ± 69.0	363.7 ± 54.9
Interleukin-17	12.9 ± 1.2	20.2 ± 3.7	11.9 ± 2.6^‡^	12.2 ± 2.7^‡^
Interleukin-18	40.1 ± 5.1	60.9 ± 8.8^*^	40.2 ± 8.5	33.2 ± 6.3^‡^
IP-10	153.0 ± 30.4	137.1 ± 39.8	115.2 ± 28.3	109.2 ± 16.5^‡^
LIX/ CXCL5	ND	68.3 (1)	ND	ND
Leptin	86.5 ± 18.3	110.0 ± 17.9	63.3 ± 14.6	64.8 ± 12.4
MCP-1	143.0 ± 30.6	187.2 ± 33.5	127.1 ± 53.2 (4)	155.4 ± 14.8 (5)
MIP-1 alpha	ND	ND	ND	ND
MIP-2	93.6 ± 6.5	110.2 ± 11.2	63.7 ± 7.6^*^^‡^	66.6 ± 7.4^*^^‡^
RANTES/ CCL5	33.3 ± 5.5	32.6 ± 6.8	22.8 ± 3.7	25.7 ± 3.9
TNF alpha	0.6 ± 0.2 (2)	1.3 ± 0.9 (2)	0.8 (1)	0.6 ± 0.2 (2)
VEGF	14.1 ± 1.6	18.3 ± 2.2	9.9 ± 1.5^‡^	10.0 ± 1.7^‡^

* p < 0.05 vs. NP;

‡ *p < 0.05 vs. RUPP. Data represent mean ± SEM. The number in parenthesis represents the number of samples with detectable concentrations. ND, none detected; EGF, epidermal growth factor; G-CSF, granulocyte colony stimulating factor; GM-CSF, granulocyte macrophage colony stimulating factor; GRO/KC/CINC, Growth-regulated oncogene/keratinocyte chemoattractant/cytokine-induced neutrophil chemoattractant; IP-10, Interferon gamma-induced protein 10; LIX, Lipopolysaccharide-induced CXC chemokine; MCP-1, monocyte chemotactic protein 1; MIP, macrophage inflammatory protein; RANTES, regulated on activation; normal T cell expressed and secreted; TNF, tumor necrosis factor; VEGF, vascular endothelial growth factor. CSF cytokine data for NP vs. RUPP has been published previously (Warrington, 2015)*.

## Discussion

The most widely used drug for the treatment of preeclampsia patients with severe symptoms is magnesium sulfate. While it is effective in preventing seizure activity, the mechanisms by which it does so are not fully known. This study tested the hypothesis that MgSO_4_ acts by protecting the brain from edema formation and by reducing the levels of cytokines in the cerebrospinal fluid. We found that while MgSO_4_ had no effect on improving blood pressure or fetal outcomes following placental ischemia, it reduced CSF protein and albumin concentration as well as the level of cytokines in placental ischemic rats and prevented placental ischemia-induced increases in brain water content.

Numerous studies have shown a beneficial role for MgSO_4_ in preventing seizures; however, there have been reports of no effects on other characteristic symptoms of preeclampsia. For example, MgSO_4_ had no effect on angiogenic factors in preeclampsia patients treated with MgSO_4_ (Vadnais et al., 2012; Eshkoli et al., 2013). Importantly, MgSO_4_ was shown to have no effect on reducing blood pressure in a rat model of preeclampsia induced by lipopolysaccharide injection (Huang et al., 2014) or the model of acute hypertension in pregnant rats (Euser et al., 2008). The current study showed no change in placental weight following placental ischemia but a significant reduction in placental weight following placental ischemia and MgSO_4_ treatment. We do not know why MgSO_4_ would lead to reduced placental weight following placental ischemia. Taken together, these studies, along with the findings from the current study, support the concept that MgSO_4_ improves the cerebral symptoms but has minimal effects on other characteristic symptoms of preeclampsia.

Preeclampsia is characterized by decreased cardiac output and increased peripheral resistance, two factors that contribute to the hypertension observed (Guy et al., 2016). The hemodynamic changes in the placental ischemia model have been characterized in a study by Sholook et al. (2007). In that study, the authors reported a 50–60% decrease in uteroplacental blood flow with no change in blood flow to the kidney, liver, or brain, and a decrease in blood flow to the heart. Like preeclampsia patients, placental ischemic rats also demonstrated decreased cardiac output and increased total peripheral resistance (Sholook et al., 2007). Thus, the placental ischemic (RUPP) model mimics the hemodynamic changes that occur in preeclampsia.

Because (pre) eclampsia patients sometimes present with various cerebrovascular abnormalities, including cerebral edema, this study assessed changes in brain water content in rats subjected to placental ischemia with MgSO_4_ treatment. As shown previously, brain water content increased in response to placental ischemia in the anterior cerebrum (Warrington et al., 2014, 2015). Placental ischemia-induced increase in brain water content was prevented by MgSO_4_ treatment. The increase in brain water content in response to placental ischemia is consistent with studies showing edema in preeclampsia patients (Mitas and Rogulski, 2012), and the placental ischemic model (Ryan et al., 2011; Warrington et al., 2014). It should be noted that a recent paper reported decreased brain water content in the posterior cerebrum of rats subjected to placental ischemia plus a high cholesterol diet (Johnson et al., 2014). While this finding appears contradictory to our current findings, the placental ischemic model seems to be protected from posterior cerebral edema and this study, along with previously published studies, demonstrate that the anterior cerebrum is more susceptible to cerebrovascular abnormalities following placental ischemia (Warrington et al., 2014, 2015). Additionally a key difference between the study by Johnson et al. (2014) and this one is that the brain water content was measured following seizures in the paper by Johnson et al. (2014) while the current study measured brain water content following placental ischemia alone without additional interventions.

While we do not know why the anterior cerebrum is more susceptible to edema formation following placental ischemia, we have shown that BBB permeability also increases in the anterior cerebrum but not the posterior cerebrum (Warrington et al., 2014) supporting the idea that increases in BBB permeability in the anterior cerebrum contributes to the increased brain water content. It is thought that vasogenic edema (through BBB disruption) is the primary form of edema in preeclampsia (Cipolla, 2007). We have not determined whether there are ultrastructural changes at the BBB following placental ischemia; however, we showed no changes in the expression of claudin-1, occludin, or zonular occludens 1 in the anterior brain following placental ischemia, suggesting that reductions in the expression of tight junction proteins did not contribute to edema formation following placental ischemia (Warrington et al., 2014).

It is possible that cytotoxic edema (cell swelling) could contribute to anterior cerebral edema following placental ischemia. Indeed, a recent study showed that traumatic brain injury-induced cerebral edema is associated with increased expression of aquaporin 4 (AQP4) and vasopressin 1a receptor (Marmarou et al., 2014) and astrocytic swelling. We showed previously that protein expression of AQP4 is increased in the posterior cerebrum but unchanged in the anterior cerebrum. We hypothesized that increased AQP4 protein expression in the posterior cerebrum is an indicator of increased edema resolution. Because we have not determined whether astrocyte morphology changes in response to placental ischemia, we are unable to determine whether the edema formation occurs as a result of astrocyte swelling as well. Thus, MgSO_4_ could contribute to resolution of placental ischemia-induced increases in brain water content through changes in AQP4 and vasopressin receptor expression by astrocytes, a question that will be answered in future studies.

While the mechanisms by which MgSO_4_ exerts its effects are not well understood, some studies have suggested potential mechanisms ranging from vasodilation to reduced blood-brain barrier permeability. There is evidence that in the cerebral vasculature, MgSO_4_ acts as a vasodilator (Altura et al., 1987; Perales et al., 1991; Belfort and Moise, 1992; Belfort et al., 1993; Naidu et al., 1996; Euser and Cipolla, 2005), thus reducing the increased pressure within the vasculature. MgSO_4_ may offer neuroprotection through decreasing BBB permeability. Indeed, MgSO_4_ has been shown to decrease BBB permeability following traumatic brain injury (Esen et al., 2003), septic encephalopathy (Esen et al., 2005), and hypoglycemia (Kaya et al., 2001). MgSO_4_ has also been shown to reduce cerebral edema formation following brain injury (Esen et al., 2003). Furthermore, MgSO4 treatment reduced permeability to Evans blue dye in pregnant rats subjected to acute hypertension (Euser et al., 2008) in both anterior and posterior cerebrum. In the current study, we did not measure changes in BBB permeability to Evans blue dye or other similar molecules neither did we measure changes in the ultrastructure of the tight junctions. We, however, determined changes in brain water content to determine cerebral edema formation, a consequence of increases in BBB permeability. We report that MgSO_4_ reduces brain water content possibly by reducing BBB permeability, previously shown to be increased in the rat placental ischemic model of preeclampsia (Warrington et al., 2014). Future studies will assess whether placental ischemia induces damage to the choroidal ependymal cells or the endothelial tight junctions and whether MgSO_4_ reverses that damage.

It is widely accepted that cytokines can influence brain signaling and can contribute to changes in cerebrovascular function as well as neuronal excitability. One potential mechanism by which MgSO_4_ could have protective effects in the brain could be through decreasing levels of cytokines/chemokines. Both preeclampsia patients and the rat model of placental ischemia are characterized by increased levels of circulating and placental inflammatory cytokines. Importantly, we recently showed that CSF levels of the cytokines/chemokines eotaxin, IL-2, IL-17, and IL-18 are increased in the placental ischemic rat (Warrington, 2015). We have extended these findings to determine whether MgSO_4_ treatment would lead to a reduction in levels of CSF cytokines/chemokines. Our results indicate that 9 out of 27 cytokines/chemokines (IL-17A, IL-18, eotaxin, fractalkine, interferon gamma, IL-2, GRO/KC/CINC, macrophage inflammatory protein 2 (MIP-2), and vascular endothelial growth factor (VEGF)) were reduced by MgSO_4_ treatment in normal pregnant or placental ischemic rats.

Increased levels of IL-17 (Martínez-García et al., 2011; Toldi et al., 2011; Darmochwal-Kolarz et al., 2012) and IL-18 (Huang et al., 2005; Seol et al., 2009; El-Kabarity and Naguib, 2011) have been reported in preeclampsia patients. A direct role for IL-17 in mediating hypertension during pregnancy was demonstrated using animal studies where infusion of IL-17 into the pregnant rat increased mean arterial pressure and oxidative stress (Dhillion et al., 2012) while reducing IL-17 in placental ischemic rats attenuates blood pressure and oxidative stress (Cornelius et al., 2013). While there are no reports of changes in IL-17 levels following eclamptic seizures, there are reports that IL-17 is increased in the circulation of epilepsy patients in between seizures or following seizures and IL-17 levels was correlated with seizure frequency and severity in epilepsy patients (Mao et al., 2013). CSF IL-17 levels were also elevated in patients between seizure episodes (Mao et al., 2013) and were correlated with glutamate levels and BBB disruption in a group of multiple sclerosis patients (Kostic et al., 2014). Furthermore, IL-17 has been shown to induce BBB disruption through the production of reactive oxygen species and down-regulation of Occludin (a tight junction protein) (Huppert et al., 2010). Thus, the current findings support the hypothesis that MgSO_4_ offers protection against BBB and blood-CSF-barrier permeability by decreasing IL-17 levels in the CSF. Further studies are required to directly test this hypothesis.

Eotaxin (CCL11) and fractalkine are chemoattractants. Eotaxin is released by activated astrocytes and act on microglia to promote glutamate-induced neurotoxicity (Parajuli et al., 2015) while fractalkine is released from neurons and glia and act mainly on microglia. While there are no studies reporting changes in eotaxin levels during preeclampsia, the current study shows increased CSF eotaxin following placental ischemia and reduced levels following MgSO_4_ treatment. It is possible that placental ischemia-induced increases in CSF eotaxin may indicate increased glutamate activity and neurotoxicity and the MgSO_4_ protection against seizure may act through reduced eotaxin levels. Future studies will determine whether placental ischemia is associated with increased glutamate signaling. A recent study showed that placental fractalkine levels are increased in severe early preeclampsia patients (Siwetz et al., 2015). Because fractalkine can reduce GABAergic function in pyramidal neurons in patients with mesial temporal lobe epilepsy (Roseti et al., 2013), it is possible that MgSO_4_ may exert its anti-seizure effects through reduced fractalkine levels. This possibility was not tested in the current study and will be the subject of future studies.

The pro-inflammatory cytokine, interferon gamma (Ozkan et al., 2014; Yang et al., 2014) has been reported to be increased in the circulation (plasma and serum) of preeclampsia patients. Additionally, patients with temporal lobe epilepsy have also been reported to have increased levels of interferon gamma (Vieira et al., 2016). However, the effects of MgSO_4_ treatment on these cytokines have not been assessed. The current study shows that interferon gamma is reduced following MgSO_4_ treatment, thereby reducing the inflammatory status and offering neuroprotection. There are no studies showing a relationship between MIP-2 levels in preeclampsia, eclampsia, or epileptic patients. However, the finding that MIP-2 is reduced following MgSO_4_ treatment suggests that MIP-2 may be involved in increased seizure susceptibility and its role in mediating increased seizure susceptibility will be explored in future studies.

We found a significant reduction in cerebrospinal fluid VEGF levels following MgSO_4_ treatment but no change with placental ischemia alone. VEGF is a pro-angiogenic factor, released in conditions of tissue hypoxia (Pugh and Ratcliffe, 2003; Liao and Johnson, 2007; Denko, 2008). VEGF is also associated with increased vascular permeability and has been shown to be important during cerebral edema formation (Huang et al., 2016). Further studies are required to assess whether decreases in CSF VEGF after chronic MgSO_4_ treatment could indicate reduced tissue levels of VEGF, resolution of tissue hypoxia, and whether VEGF is important for placental ischemia-induced cerebral edema formation, and MgSO_4_-induced resolution of cerebral edema. While one study showed increases in circulating TNFα in a rat model of preeclampsia and decreases in TNFα following MgSO_4_ treatment (Huang et al., 2014), we found no differences in the level of CSF TNFα following placental ischemia or MgSO_4_ treatment possibly due to the fact that the majority of the samples were below detectable range.

The recently described glymphatic system describes a role for the CSF in collecting products from the extracellular space into the CSF (Iliff and Nedergaard, 2013; Mendelsohn and Larrick, 2013; Plog et al., 2015) by neuroglia. Indeed, a recent study demonstrated increased levels of activated microglia in placental ischemic rats fed a high cholesterol diet and that MgSO_4_ reverses the increase in activated microglia (Johnson et al., 2014). While the current study is unable to establish the source of the CSF cytokines/chemokines, the increases in CSF cytokines following placental ischemia could indicate increased inflammation at the tissue level and increased numbers of activated microglia while the reduction in CSF cytokines following MgSO_4_ treatment could indicate reduced tissue inflammation and reduced number of activated microglia. This will be tested in future studies. It is also possible that increases in CSF cytokines may represent increased clearance of tissue inflammation. Therefore, changes in the glymphatic system following placental ischemia and MgSO_4_ treatment will be determined in future studies.

The current study represents a hypothesis generating study that presents several potential mechanisms by which MgSO_4_ offers neuroprotection following placental ischemia, relevant for the clinical condition of preeclampsia. We showed that MgSO_4_ prevents placental ischemia-induced increases in brain water content and CSF cytokines/chemokines. However, whether the observed differences translate to neuroprotection is not known. Based on our findings, MgSO_4_ therapy seems to preferentially protect the brain from cerebrovascular insults in a model of placental ischemia that closely mimics the clinical condition of (pre) eclampsia. Specifically, MgSO_4_ protects against cerebral edema formation and CSF inflammation and may be a mechanism for the prevention of seizures or the progression from preeclampsia to eclampsia. Because this study utilized the chronic infusion of MgSO_4_ (5 days) that commenced from the initiation of placental ischemia, we are unable to discuss whether acute treatment will have similar effects. Also, whether chronic infusion of MgSO_4_ can lower seizure threshold in rats subjected to placental ischemia alone is not known.

## Author contributions

JW, designed the study. LZ and JW, collected and analyzed data. LZ and JW, drafted the manuscript. LZ and JW approved manuscript.

## Funding

This study was supported by the National Institute of General Medical Sciences of the National Institutes of Health under Award Number P20GM104357 and the National Heart, Lung, And Blood Institute of the National Institutes of Health under Award Number P01HL051971, K99HL129192, and the American Heart Association grant: 13POST16240000. The content is solely the responsibility of the authors and does not necessarily represent the official views of the National Institutes of Health or American Heart Association.

### Conflict of interest statement

The authors declare that the research was conducted in the absence of any commercial or financial relationships that could be construed as a potential conflict of interest.
